# Effect of technology innovation and spillovers on the carbon intensity of human well-being

**DOI:** 10.1186/s40064-016-1984-0

**Published:** 2016-03-18

**Authors:** Jifang Feng, Jianhong Yuan

**Affiliations:** School of Marxism, Southeast University, Nanjing, 211189 China

**Keywords:** Carbon intensity, Human well-being, Technology innovation, Technology spillovers, Sustainability

## Abstract

In order to enhance sustainability, it is necessary to reduce the carbon intensity of human well-being (CIWB). In this paper, we analyze the impact of technology innovation and spillovers on CIWB using panel data of 30 provinces in China from 2005 to 2010. We find that increasing research and development (R&D) intensity and interregional R&D spillovers can decrease CIWB; R&D intensity has a nonlinear effect on CIWB without incorporating interregional R&D spillovers; economic development has positive effect on CIWB, while manufacturing has negative effect on CIWB.

## Background

In order to improve human well-being, we make use of the biophysical environment to promote economic development. This results in pressure on the environment in various ways, including alteration of biogeochemical cycles, appropriation of biomass and transformation of land cover (Levy and Morel 2012). Therefore it is necessary to balance ecological and environmental concerns with human well-being and economic development, which is the pathway to sustainable development.

The goal of sustainability is to minimize environmental impacts and maximize human well-being (Prescott-Allen 2001; Dietz et al. 2009). There is a lot of literature on the relationship between environmental consumption and well-being (e.g., Mazur and Rosa 1974; Dietz et al. 2009, 2012; Knight and Rosa 2009; Venhoeven et al. 2013; Jorgenson et al. 2014). Knight and Rosa (2011) point out that research on the relationship between environmental consumption and well-being leads to a key refinement, the idea of sustainability conceptualized as environmental efficiency of well-being. Based on this idea, sustainability is measured as the ecological intensity of human well-being (EIWB; e.g., Knight and Rosa 2011; Dietz et al. 2012; Jorgenson and Dietz 2015). Several recent studies have begun to analyze what social factors influence EIWB. For example, Knight and Rosa (2011) test the effects of climate, political, economic, and social factors on EIWB with a sample of 105 countries; Dietz et al. (2012) and Jorgenson and Dietz (2015) analyze the relationship between economic development and EIWB.

Built on the broader EIWB approach in structural human ecology (Dietz et al. 2009, 2012), the relationship between carbon emissions and human well-being is referred to by Jorgenson (2014) as the carbon intensity of human well-being (CIWB), which is typically operationalized as a ratio between carbon emissions per capita and an established measure of human well-being. Recent studies focus on the question of if reducing CIWB is a potential pathway towards greater sustainability, how we can successfully achieve it. For example, Jorgenson (2014) examines the effect of economic development on CIWB; Jorgenson (2015) focuses on analyzing the relationship between CIWB and domestic income inequality.

The identification of other socioeconomic conditions that might influence CIWB could steer humanity in a more sustainable direction. In general, technical change is at the heart of environmental improvements and economic development (Sterner and Turnheim 2009). In order to enhance our understanding of CIWB and the extent to which the regions’ CIWB is influenced by technical changes, in this paper we investigate the effect of technology innovation and spillovers on CIWB using panel data of China’s 30 provinces from 2005 to 2010.

## Materials and research methods

### The sample

Mainland China has 31 provincial-level districts. Since some data of Tibet are missing, the rest provinces are chosen as the sample. In this paper we analyze a perfectly balanced dataset that consists of annual observations from 2005 to 2010 for the sample of 30 provinces. This results in a sample of 180 total observations. All data used in this paper are from officially published statistics in China, including China Statistical Yearbook and China Energy Statistical Yearbook, where the missing data about average life expectancy at birth are obtained by linear interpolation method.

### Measure of CIWB

The ecological intensity of human well-being is a ratio between a measure of environmental stress and that of human well-being. Recent analyses use the ecological footprint or greenhouse gas emissions to measure environmental stress, and average life expectancy at birth to measure human well-being (Dietz et al. 2012; Jorgenson and Dietz 2015; Jorgenson 2014). In this paper, we employ the method introduced by Jorgenson (2014): carbon dioxide emissions per capita divided by average life expectancy at birth. Following his method, we constrain the coefficient of variation (standard deviation/mean) of the numerator and the denominator to be equal by adding a constant to the numerator, which shifts the mean without changing the variance. Thus, the measure of CIWB we employ is as follows.

1 $$\begin{aligned} CIWB=[(\text{CO}_{2}\text{PC}+33.5)/LE]\times 100 \end{aligned}$$

where $\text{CO}_{2}\text{PC}$ is the carbon dioxide emissions per capita in metric tons; *LE* is the average life expectancy at birth in years; the number 33.5 is calculated from the data we employed to make the coefficient of variation of the carbon dioxide emissions per capita and the average life expectancy at birth be equal; the number 100 is the scale of the ratio. In this paper, we adopt the guidelines of IPCC (2006) to calculate carbon dioxide emissions.

According to IPCC (2006), carbon dioxide emission in this paper is calculated via the following equation.

2 $$\begin{aligned} CI_{it}=\sum \limits _{r=1}^{m}E_{itr}\times \lambda _{r}\times O_{r}\times \frac{44}{12} \end{aligned}$$

where $CI_{it}$ is the carbon dioxide emission of province *i* in year *t*, and $E_{itr}$ is the consumption of fossil energy *r* of province *i* in year *t*. $\lambda _{r}$ [unit: kgc/kg (km$^{3}$)] is the carbon emission coefficient of fossil energy *r*, and is calculated as the product of the default carbon content and the average net calorific value, where the data are from IPCC (2006) and the China Energy Statistical Yearbook. $O_{r}$ is the carbon oxidation rate of fossil energy *r*, and is set at the default value of 1. In this paper we consider 8 kinds of fossil energy (m = 8), namely, raw coal, coke, crude oil, gasoline, kerosene, diesel oil, fuel oil and natural gas, with the unit of the first 7 kinds of fossil energy being KJ/kg and the unit of the last one being kg/m$^{3}$.

### Measures of technology innovation and spillovers

On the one hand, technical change plays a significantly important role in improving environmental performance (Jaffe et al. 2003), which can be achieved through R&D activity and interregional knowledge spillovers. For example, Fisher-Vanden et al. (2004) find R&D expenditure to be one of the principal drivers of China’s declining energy intensity; some studies find that the interregional knowledge spillovers can boost local innovation (e.g., Bottazzi and Peri 2003; Moreno et al. 2005). On the other hand, it is also beneficial to human well-being (Kavetsos and Koutroumpis 2011; Graham and Nikolova 2013). Therefore, it is important to address the advantages of technical change to society.

Technology innovation and spillovers are recognized as the main determinants of technology progress (Coe and Helpman 1995; Keller 2004). Analyzing determinants of technology innovation has developed inside the knowledge production function framework (Griliches 1979). Audretsch and Feldman (2004) demonstrate that the relationship between innovative outputs and innovative inputs (R&D inputs) is stronger. In addition, geographic proximity to innovation producers can favor knowledge spillovers within the region, proximity to other innovative regions can boost local innovation (Cabrer-Borras and Serrano-Domingo 2007). Therefore, in this paper we choose technology innovation and spillovers as independent variables, and analyze their effects on CIWB.

Similar to Yang et al. (2014), in this paper we adopt R&D intensity to measure technological innovation. Some studies on technological change support the idea that the interregional knowledge spillovers can boost local innovation (e.g., Bottazzi and Peri 2003; Moreno et al. 2005). And R&D spillovers are one of the main forms of interregional knowledge spillovers (Yang et al. 2014). Therefore, in this paper we use interregional R&D spillovers to measure technology spillovers.

Following Coe and Helpman (1995) and Yang et al. (2014), we use weighted average of R&D intensity of neighboring regions to reflect interregional R&D spillovers. Thus, the following formula is derived.

3 $$\begin{aligned} TS_{it}=\sum \limits _{j=1}^{k}w_{ij}\times RD_{jt} \end{aligned}$$

where *TS* denotes technology spillover; $w_{ij}$ is the spatial weight to reflect the relationship between province *i* and province *j*. If province *i* and province *j* are neighbors, $w_{ij}=1$; otherwise $w_{ij}=0$. RD is the R&D intensity, which is computed as R&D stock divided by GDP. The unit of measurement for GDP is million yuan RMB, and is in current price. The R&D stock $K_{it}$ can be calculated as the accumulation of R&D expenditures minus depreciation (Coe and Helpman 1995).

4 $$\begin{aligned} K_{it}=(1-\delta )K_{i,t-1}+I_{it} \end{aligned}$$

where $\delta$ is the depreciation rate and is set at 15 %; $I_{it}$ is the R&D investment of province *i* in year *t*. The base year (2005) can be calculated through the following formula.

5 $$\begin{aligned} K_{i,2005}=\frac{I_{i,2006}}{r_{2005-2010}+\delta } \end{aligned}$$

where $r_{2005-2010}$ is the average growth rate of R&D expenditures from 2005 to 2010.

### Model specification

In this paper, two variables are chosen as control variables, namely GDP per capita and manufacturing. Previous work on CIWB, particularly Jorgenson (2014), finds economic development affects CIWB. Therefore GDP per capita is chosen as one control variable. On the one hand, it is often found that relative levels of manufacturing increase energy consumption (Clark et al. 2010), which increase carbon dioxide emissions. On the other hand, the impacts of manufacturing on life expectancy or other aspects of human well-being remain understudied and under-theorized in macro-comparative contexts (Jorgenson et al. 2014). Hence, manufacturing is chosen as the other control variable, which is measured as the ratio of manufacturing output to GDP in this paper.

In order to investigate the effect of technology innovation and spillovers on CIWB, based on the above analysis, the following double log model is derived.

6 $$\begin{aligned} lnCIWB_{it}=\phi _{i}+\varphi _{t}+\alpha _{1}lnTI_{it}+\alpha _{2}lnTS_{it}+\alpha _{3}lnGDPPC_{it}+\alpha _{4}lnM_{it}+\varepsilon _{it} \end{aligned}$$

where subscript *i* denotes province *i* and subscript *t* denotes year *t*. $CIWB_{it}$ indicates the carbon intensity of human well-being of province *i* in year *t*. $\phi _{i}$ and $\varphi _{t}$ are the regional effect and the year effect respectively. $\varepsilon$ is error term; *TI* is technology innovation; *TS* is technology spillover; *GDPPC* is GDP per capita; and *M* is manufacturing.

## Results

Table 1 presents the description statistics of the variables included in the analysis. Since Hausman test and Redundant Fixed Effects Tests are both significant at the level 1 %, the fixed effects model is chosen and the random effects model is rejected in the following estimation. In addition, through serial correlation and heteroskedasticity tests, it is detected that both serial correlation and heteroskedasticity are present. A feasible solution to overcome this problem is to use the panel-corrected standard error (PCSE) estimates, which is proposed by Beck and Katz (1995), as PCSE is panel-corrected standard errors estimation with heteroskedastic and AR(1) disturbances. Therefore, in this paper we use PCSE to estimate Eq. (6). Results are presented in Columns (1), (2), (3) and (4) in Table 2.

**Table 1:** Description statistics of variables

Symbol	Mean	Standard deviation	Skewness	Kurtosis
*lnCIWB*	3.8725	0.0447	0.7969	2.7474
*lnTI*	−4.1036	0.9258	−0.3396	3.0734
*lnTS*	−2.6202	0.8497	−0.5043	2.6556
*lnGDPPC*	0.4215	0.5044	0.6809	2.8826
*lnM*	−0.9131	0.2257	−1.7643	6.5171

**Table 2:** Results of models obtained from PCSE estimation

No.	(1)	(2)	(3)	(4)
*lnGDPPC*	0.0400***	0.0213***	0.0216***	0.0413***
	(0.0034)	(0.0043)	(0.0042)	(0.0031)
*lnM*	−0.0040*	−0.0098***	−0.0096***	−0.0060*
	(0.0023)	(0.0024)	(0.0025)	(0.0033)
*lnTI*	−0.0048***	−0.0215***	−0.0212***	−0.0027**
	(0.0002)	(0.0025)	(0.0027)	(0.0013)
*lnTS*			−0.00003	−0.0020*
			(0.0009)	(0.0012)
$lnTI^{2}$		−0.0020***	−0.0019***	
		(0.0003)	(0.0003)	
$R^{2}$	0.9922	0.9923	0.9927	0.9927
Adjusted $R^{2}$	0.9906	0.9868	0.9910	0.9910

(1) Values in parentheses are standard errors. (2) $$*, **$$ and $$***$$ denote significance at 10, 5, and 1 $$\%$$ level respectively. (3) PCSE is panel-corrected standard errors estimation with heteroskedastic and AR(1) disturbances

For the estimated coefficients in Columns (1) and (2), technology innovation (*lnTI*) and its squared term ($lnTI^{2}$) are significant and negative. This means that there exists a nonlinear effect of technology innovation on CIWB. Comparing the estimated coefficient of *lnTI* with that of $lnTI^{2}$, we can find that technology innovation’s linear effect is stronger than its nonlinear effect. The implication of this result is that R&D activity is beneficial for the decrease in CIWB, where the effect of R&D activity depends on the level of R&D intensity. A possible reason is that provinces with high R&D intensity level have better technology for controlling carbon dioxide emissions and improving human well-being. The nonlinear effect of technology innovation is also found by Yang et al. (2014), when they analyze the effect of technology innovation on industrial CO$_{2}$ intensity.

Results in Columns (1) and (2) are estimated without the consideration of the effect of technology spillovers (*lnTS*). While results in Columns (3) and (4) are estimated with the consideration of the effect of technology spillovers. From Columns (3) and (4), it can be seen that term *lnTS* is not significant when term $lnTI^{2}$ is included, but it is significant when term $lnTI^{2}$ is not added. Therefore, technology innovation and spillovers have inhibitory effects on CIWB when the nonlinear effect is not considered. This means that raising R&D intensity and interregional R&D spillovers increases the beneficial effect on CIWB. A possible reason for this is that provinces with high R&D intensity level not only have better technology for controlling carbon dioxide emissions and improving human well-being, but also have capabilities to adopt and absorb advanced technologies from neighboring provinces.

For the estimated coefficients in Columns (1)–(4), economic development (*lnGDPPC*) and manufacturing (*lnM*) are significant. We find that economic development increases CIWB. In fact, empirical results suggest that carbon dioxide emissions increase with the increase in GDP per capita (Fei et al. 2011; Alkhathlan and Javid 2013). For example, Fei et al. (2011) examine the causal relationship between CO$_{2}$ emissions, energy consumption and economic growth for 30 provinces of mainland China from 1985 to 2007. The long run positive cointegrated relationship of their paper showed that if GDP per capita increases by $1~\%$, energy consumption will increase by $0.50~\%$ approximately, while CO$_{2}$ emissions will increase by $0.43~\%$. Therefore, a possible reason for the effect of economic development on CIWB is that economic development and carbon dioxide emissions are positively related, and economic development and human well-being are positively related. But the effect of economic development on carbon dioxide emissions is larger than that on human well-being.

In this paper, we find the above effect of economic development on CIWB from the regional level. In fact, this result is also found by Jorgenson (2014) from the national level. Jorgenson (2014) examines how the effect of economic development on CIWB has changed since 1970 for 106 countries, and finds that economic development increases CIWB for nations in Asia and South and Central America. For manufacturing, we find that it has a negative effect on CIWB. The development of manufacturing leads to an increase in the consumption of carbon-intensive fuels (Kander 2002; Wang et al. 2005; Wu et al. 2005; Aslan et al. 2013). And there is a positive relation between energy consumption and carbon dioxide emissions (Aslan et al. 2013). Therefore, the development of manufacturing increases carbon dioxide emissions. Hence, a possible explanation for the negative effect of manufacturing on CIWB is that the positive effect of manufacturing on human well-being overweighs its positive effect of carbon dioxide emission.

## Conclusion

Reducing the carbon intensity of human well-being is a potential pathway towards greater sustainability. It is important to identify socioeconomic conditions that might reduce the carbon intensity of human well-being. In this paper, we investigate the effect of technology innovation and spillovers on the carbon intensity of human well-being, using a sample of 30 provinces in China from 2005 to 2010.

In our study, technology innovation and spillovers are indicated by R&D intensity and interregional R&D spillovers respectively. When we conduct empirical analysis, economic development and manufacturing are chosen as control variables. The model constructed in this paper is analyzed empirically by PCSE estimation. Through empirical analysis, we find that increasing R&D intensity and interregional R&D spillovers can decrease the carbon intensity of human well-being. It is found that R&D intensity has a nonlinear effect on the carbon intensity of human well-being without the consideration of interregional R&D spillovers. In addition, empirical results reveal that economic development has a positive effect on the carbon intensity of human well-being, while manufacturing has an opposite effect on the carbon intensity of human well-being.

Technology innovation is the source of the creation of new technology for controlling environmental pollution and improving human well-being, and it is also the precondition for knowledge spillovers. In order to achieve sustainable development in China, the above results suggest that it is necessary to strength policies on building innovation capacity and policies on promoting knowledge spillovers through interregional cooperation and technology transfer simultaneously.
